# Carbon Nanotubes’ Effect on Mitochondrial Oxygen Flux Dynamics: Polarography Experimental Study and Machine Learning Models using Star Graph Trace Invariants of Raman Spectra

**DOI:** 10.3390/nano7110386

**Published:** 2017-11-11

**Authors:** Michael González-Durruthy, Jose M. Monserrat, Bakhtiyor Rasulev, Gerardo M. Casañola-Martín, José María Barreiro Sorrivas, Sergio Paraíso-Medina, Víctor Maojo, Humberto González-Díaz, Alejandro Pazos, Cristian R. Munteanu

**Affiliations:** 1 Institute of Biological Science (ICB), Federal University of Rio Grande, Rio Grande, RS 96270-900, Brazil; gonzalezdurruthy.furg@gmail.com; 2 Department of Coatings and Polymeric Materials, North Dakota State University (NDSU), Fargo, ND 58102, USA; bakhtiyor.rasulev@ndsu.edu; 3 Department of Systems and Computer Engineering, Carleton University, Ottawa, ON K1S 5B6, Canada; gmaikelc@gmail.com; 4 Computer Science School (ETSIINF), Polytechnic University of Madrid (UPM), Calle de losCiruelos, Boadilla del Monte, 28660 Madrid, Spain; jmbarreiro@fi.upm.es; 5 Biomedical Informatics Group, Artificial Intelligence Department, Polytechnic University of Madrid, Calle de los Ciruelos, Boadilla del Monte, 28660 Madrid, Spain; sparaiso@infomed.dia.fi.upm.es (S.P.-M.), vmaojo@fi.upm.es (V.M.); 6 Department of Organic Chemistry II, University of the Basque Country UPV/EHU, 48940 Leioa, Biscay, Spain; gonzalezdiazh@yahoo.es; 7 IKERBASQUE, Basque Foundation for Science, 48011 Bilbao, Biscay, Spain; 8 INIBIC Institute of Biomedical Research, CHUAC, UDC, 15006 Coruña, Spain; apazos@udc.es; 9 RNASA-IMEDIR, Computer Sciences Faculty, University of Coruña, 15071 Coruña, Spain

**Keywords:** carbon nanotubes, cytotoxicity, mitochondria oxygen mass flux, Raman spectroscopy, graph theory, spectral moments

## Abstract

This study presents the impact of carbon nanotubes (CNTs) on mitochondrial oxygen mass flux (*J_m_*) under three experimental conditions. New experimental results and a new methodology are reported for the first time and they are based on CNT Raman spectra star graph transform (spectral moments) and perturbation theory. The experimental measures of *J_m_* showed that no tested CNT family can inhibit the oxygen consumption profiles of mitochondria. The best model for the prediction of *J_m_* for other CNTs was provided by random forest using eight features, obtaining test R-squared (*R*^2^) of 0.863 and test root-mean-square error (RMSE) of 0.0461. The results demonstrate the capability of encoding CNT information into spectral moments of the Raman star graphs (SG) transform with a potential applicability as predictive tools in nanotechnology and material risk assessments.

## 1. Introduction

Carbon nanotubes (CNTs) have attracted great interest for their promising applications in the fields of biomaterials and nano-biotechnology. Therefore, the evaluation of their toxicity in biological systems is a goal of major importance for the biomaterial sciences. Currently, evidence has been accumulating regarding the CNT toxicity associated with mitochondrial dysfunction and apoptosis [1]. Some in vitro studies have demonstrated that CNTs exhibit cytotoxicity after their accumulation in the mitochondria matrix and/or by affecting the function of mitochondrial respiratory complexes of the inner membrane [2,3].

However, it is still not known which bio-energetic mechanisms (the inhibition of adenosine diphosphate/adenosine triphosphate (ADP/ATP)-transport, uncoupling effects on oxidative phosphorylation, the induction of mitochondrial permeability transition pores, etc.) are responsible for the initiation of CNT mitochondrial damage [4,5,6,7,8,9]. Particularly, the study of mitochondrial dysfunction based on perturbations of the mitochondrial oxygen mass flux induced by CNTs could be decisive for the in vitro prediction of the no-observed-adverse-effect level (NOAEL) [10] and the evaluation of the selective nanotoxicity (mitochondrial channel nanotoxicity) towards potential biomedical applications in precision medicine. Several scientific reports highlight a major impact of adverse/toxic effects induced by CNT on critical mitochondrial components due to a peculiar mitotropic behavior. Mitochondrial oxygen mass flux regulates the mitochondrial volume linked to the Ca^2+^ induction of mitochondrial permeability transition pores (MPTP) and also to the increase of the mitochondrial reactive oxygen species (ROS-levels) based on the partial reduction of molecular oxygen in the mitochondrial respiratory complexes (complex IV). The latter have been extensively characterized and associated with several chronic pathological processes, such as neurodegenerative diseases (Alzheimer, Parkinson, Epilepsy), cardiac ischemia, and cancer. These diseases have currently high levels of morbidity and mortality, and mitochondrial dysfunction based on oxygen mass flux mechanisms has been indirectly or directly involved.

On the other hand, Raman spectroscopy provides information on the chemical fingerprints of molecules, biomolecular systems and nanoscale structures: DNA [11], proteins [12], antibodies [13] and CNTs [14]. In addition, the presence of the G band (1580 cm^−1^) in the Raman spectra of SWCNTs has been corroborated in mitochondria associated with incipient colloid–osmotic swelling or the induction of mitochondrial permeability transition pores [15]. Recent studies using Raman spectroscopy and polarographic methods have shown that CNTs alter cytochrome c electron transfer and modulate mitochondrial function at a critical concentration of 10 µg/mL [16]. Previous quantitative structure–activity relationship (QSAR) models of CNTs in terms of mitochondrial respiratory function have been reported [10]. However, there are only few studies about the relationship between a CNT’s physicochemical parameters from an oxygen mass flux perspective.

In principle, the Raman spectra signals of different CNTs can be used as inputs for machine learning (ML) methods to predict a dose–effect relationship for the biological properties of CNTs. Nevertheless, the Raman spectra of CNTs have many peaks (>1000 points), making statistical analysis still possible, but somewhat difficult. A possibility for this kind of signal is to compress them into another series of numerical parameters that quantify useful structural information on all the spectra. In a previous work, the star graphs (SG) of the Raman spectra of CNTs [17] were introduced. The idea is to transform the signal into a network with star graph (SG) topology. Next, different invariants can be calculated from the adjacency matrix associated with this SG graph representation of the spectra. Afterwards, the new invariants are supposed to contain useful information, compressed and used as input in ML experiments. Last, as a result of the ML study, predictive models are obtained, which are able to connect the Raman spectra with the biological activity under study. In fact, our group have used this scheme based on SRN transforms to model biological properties from protein sequences [18,19,20], nucleic acid sequences [21], blood protein mass spectra [22] and electroencephalogram (EEG) signals [23]. These studies have used different matrix invariants such as Markov–Shannon entropies or matrix trace invariants *Tr_k_* (also known as spectral moments) to compress the information from proteins, gene, etc. In a previous work, the research focused on how to use Markov–Shannon entropies to compress information from Raman spectra [17]. However, there is no report on the use of *Tr_k_* values in this sense.

**Figure 1:**
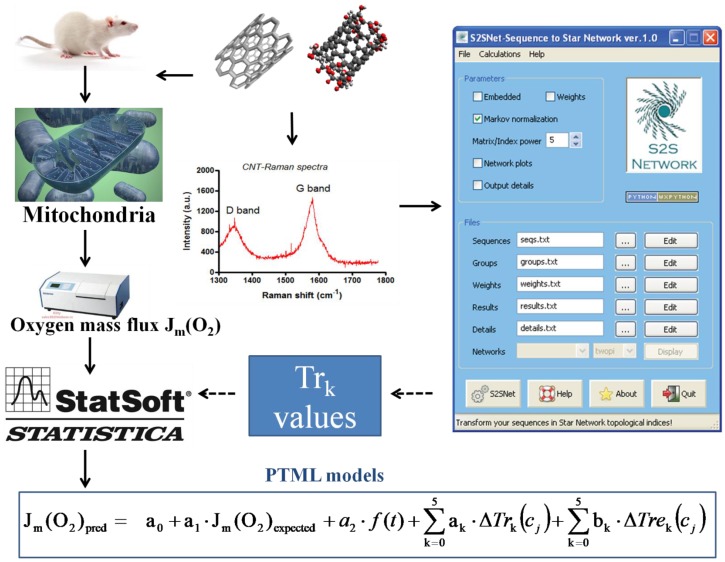
General workflow.

This work is aimed at combining experimental and computational techniques to provide a heuristic solution to the above-discussed problem. Firstly, high-resolution respirometry (HRR) polarographic (Oxygraph-2k) assays are used for the first time to address this issue. This methodology provides a quick and reproducible means to measure the rate of oxygen consumption by mitochondria isolated from different tissues using a sensitive Clark-type electrode. Simultaneously, the oxygen mass flux (O_2_ flux) in mitochondrial suspensions can be monitored depending on the time of exposure [24,25,26]. Currently, there are no precedents for this methodology applied to the evaluation of the no-observed-adverse-effect level of CNTs. In addition, a new computational model is developed for the prediction of dose–effect relationships for this property for other CNTs. In doing so, the *Tr_k_* values of the Raman spectra of CNTs are used as an input to seek a predictive model based on machine learning (ML) and perturbation theory (PT) (PTML model). PTML models are useful to predict the properties of complex molecular systems with simultaneous variations of multiple experimental boundary conditions, such as chemical reactivity, drug metabolism, vaccine peptide epitopes, metabolic networks, and micelle nanoparticles [27,28,29,30]. Figure 1 depicts a workflow scheme with the general steps of this work. The current work paves the way for the use of PTML models, polarography, and Raman spectroscopy, and for the experimental and theoretical study of other biological properties of CNTs in the future.

## 2. Results

### 2.1. Experimental Results

**Figure 2:**
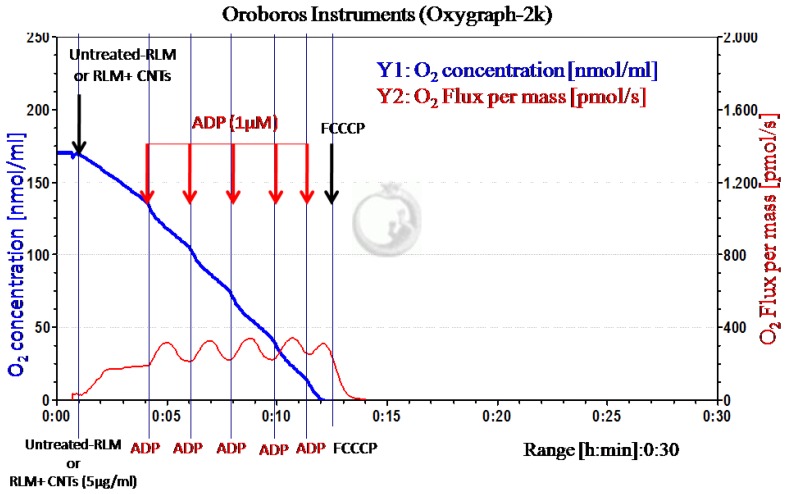
Representative profiles of the mitochondrial oxygen mass flux of isolated rat liver mitochondria (Y2: Red curve).

High-resolution respirometry (HRR) using Oroboros Instruments (Oxygraph-2k) was applied to evaluate the effects of the CNT family on the bioenergetic mitochondrial function through the measurement of oxygen mass flux after exposure with different CNTs (from CNT-1 to CNT-9). A default experimental concentration of 5 µg/mL for all CNTs was established to assess the different contribution of the remaining physical–chemical parameters in the NOAEL for the oxygen mass flux response. Figure 2 shows a representative profile of the mitochondrial oxygen mass flux of isolated rat liver mitochondria, showing the small pulses of ADP-titration associated with transient increments of O_2_ flux during the period of ATP synthesis or V3 state (ADP-dependent respiration) = ratio of mitochondrial ADP-flux (*J_m_*(ADP))/(*J_m_*(O_2_)) + mitochondrial inorganic pyrophosphate Pi-flux (*J_m_*(Pi)) for the experimental condition of untreated-rat liver mitochondria (untreated-RLM) and pre-incubation with the different CNTs (RLM + CNTs 5 µg/mL). The respiratory substrates (ADP) and the uncoupling agent carbonyl-cyanide p-trifluoromethoxyphenylhydrazone (FCCCP) were added where indicated by the arrow. In addition, in the supplementary information file, a figure that depicts a control profile of the mitochondrial oxygen mass flux of isolated RLM was included.

**Figure 3:**
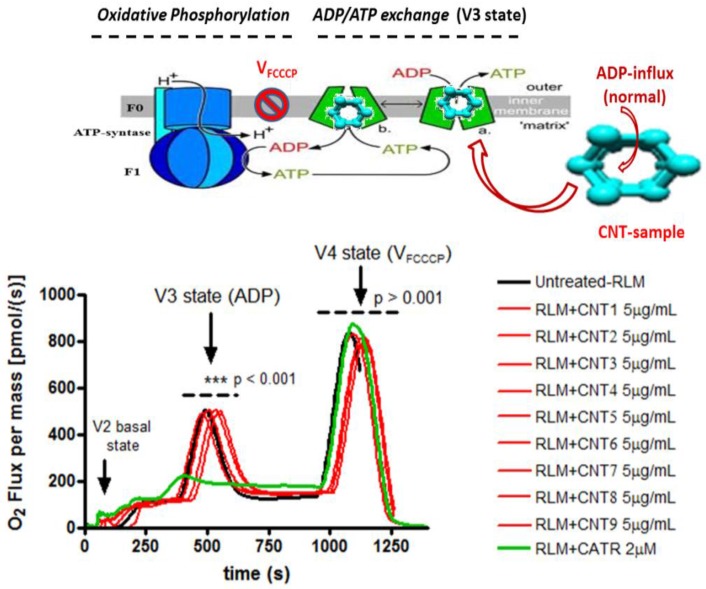
Representation of mitochondrial ADP/ATP exchange and oxidative phosphorylation. ***p is used to represent the significant statistical differences between V3 state-ADP-dependent mitochondrial O2 flux from the RLM + CNT treated groups (CNT1-9) and V3 state-ADP-dependent mitochondrial O2 flux from the RLM + Carboxyatractyloside (CATR, a specific inhibitor of ADP-mitochondrial transport).

Figure 3 shows a schematic representation of mitochondrial ADP/ATP exchange and oxidative phosphorylation based on a proposed mechanism linked to the profiles of the mitochondrial oxygen mass flux of isolated rat liver mitochondria. It shows the different states of the mitochondrial respiration V2 state (basal respiration), V3 state (ADP-dependent respiration), V4 (or VFCCCP state of ADP-independent mitochondrial respiration) in untreated rat liver mitochondria or untreated RLM (black curve), CNT-treated RLM at concentration 5 µg/mL (red curves) and treated RLM with CATR (or ADP-inhibitor) (green curve). The respiratory substrates (ADP) and the uncoupling agent (FCCCP) were added where indicated by the arrow. These results are representative for the three experiments using Oroboros Instruments (Oxygraph-2k). * *p* < 0.001 to statistical differences between CATR (or ADP-inhibitor) (green curve) and CNT-treated RLM at concentration 5 µg/mL (red curves).

### 2.2. Computational Results

The variation of the mitochondrial oxygen mass flux in the presence of CNTs was modeled using the PTML methodology based on the SG trace invariants of the Raman spectra (*Tr*_0–5_) of CNT. The RRegrs tool was adapted for calculations on an High Performance Computing (HPC) cluster and was used to test three types of regression methods: Linear Multi-regression (LM), Neural Network (NN) and Random Forest (RF). The objective of the modeling was to find the best prediction model for the mitochondrial oxygen flow in the presence of CNTs. The linear and non-linear methods were used with 10 different random data splits (75% for training–25% for test) using a custom script presented in the Figshare repository (https://doi.org/10.6084/m9.figshare.3472349). This script normalizes and splits datasets in a similar way to the RRegrs tool with the parameters *CV*types = *repeatedcv* (10-cross-validations) and default number of data splits (*iSplitTimes* = 10). After the normalization and removal of the correlated features (default RRegrs methodology *cutoff* = 0.9), eight input features remain in the final dataset. Thus, the Figshare repository presents several files: “ds2.full.Tr.csv”, the initial dataset without any filters (all features), and “ds2.corr.Tr.csv”, the final dataset after removing correlated features and using normalization. The last dataset was used with the script “CreateNormalisedSplits.R” to create 10 random splits of data as training and test subsets to test them with regression methods.

The first two are the expected values of flux, *J_m_*(O_2_)_expected_ and the duration of the experiment (*t*). The other six input variables are moving average (MA) operators, including at least one MA for each experimental condition. These MA operators are: Δ*Tr*_0_(rep), Δ*Tre*_3_(rep), Δ*Tre*_0_(CNTtype), Δ*Tre*_4_(Func.Type), Δ*Tre*_0_(Solvent), and Δ*Tre*_5_(Solvent). Please note that the MA operators Δ*Tr_k_*(*c*_j_) = *Tr_k_* − <*Tr_k_*(*c*_j_)> and Δ*Tre_k_*(*c*_j_) = *Tre_k_* − <*Tre_k_*(*c*_j_)> quantify the deviation of the *Tr_k_* or *Tre_k_* of the Raman spectra of a specific CNT from the expected values <*Tr_k_*(*c*_j_)> or <*Tre_k_*(*c*_j_)>, measured for all Raman spectra and recorded for all CNTs with the specific experimental condition (*c*_j_). The symbol *Tr_k_* refers to traces calculated from linear graphs of the Raman spectra and the symbol Tre for graphs with recurrence information embedded. Table 1 shows the minimum, maximum, and mean values for R-squared/root mean squared error of training and test subsets (*R*^2^_tr_/RMSE_tr_ and *R*^2^_ts_/RMSE_ts_) for the eight-feature dataset (10 splits). The first observation in terms of results is that the models were not over-fitted because the differences between the training and test statistics were small. Additional statistics are available online at Figshare [31] (ds2.Tr.models.xlsx).

The file ds2.Tr.models.xlsx from Figshare presents the statistics for each regression model and individual data split. In addition, minimum, maximum, and average values of the statistics are presented. The final results include the best model from an individual split and its model is saved as an R object. Thus, it is possible to directly use the best model by loading it with an R script and making any prediction. Average values of *R*^2^_ts_ and RMSE_ts_ for LM, NN and RF are presented in the same file.

## 3. Discussion

### 3.1. Discussion of Experimental Results

The results showed that all the tested CNT family did not inhibit (or affect) the profiles of oxygen mass flux in isolated rat liver mitochondria after the sequential addition of ADP intermittent pulses, which characterize the state V3 of respiration (ADP-dependent) for the untreated RLM and treated RLM with CNTs (5 µg/mL) (see Figure 2, red profile of oxygen flux). Note that for this instance, no significant differences were detected in the profiles of mitochondrial oxygen mass flux, compared with the strong inhibition of the state V3 of respiration detected for mitochondrial treated with carboxyatractyloside a specific inhibitor of ADP-mitochondrial transport (CATR-treated RLM), as shown in Figure 3 (red profile of oxygen flux). Moreover, the oxidative phosphorylation was not affected by ATP synthase, which depends on the ADP transport by ADP/ATP mitochondrial carrier between the cytosol and mitochondrial matrix under physiological normoxic conditions. The in vitro results suggest a non-inhibitory biochemical response of oxygen mass flux in isolated rat liver mitochondria. Furthermore, treated RLM with CNTs maintained the normally-induced uncoupling response of state V4 (or Vfcccp) after the addition of FCCCP 2 µM (classical uncoupling agent of the mitochondrial oxidative phosphorylation) according to an increase in the oxygen mass flux state V4 of respiration between 750 and 1250 seconds for all CNTs tested, as shown in Figure 3.

According to these results, several aspects should be considered in order to explain the relevance of CNT NOAEL in terms of mitochondrial oxygen mass flux response. Covalently functionalized CNTs (oxidized-CNTs) and/or with point defects characterized by the D band of Raman spectra (with a characteristic peak at 1350 cm^−1^) are expected to have greater biocompatibility than pristine CNTs [15]. This may be due to the ability of the OH and COOH groups of oxidized CNT or π–bond of the sp^2^ of pristine CNT wall to form several adducts with the basal oxygen-free radical [32], released by the mitochondrial complex I and III at between the V2 and V3 state of mitochondrial respiration. In this context, the non-significant respiratory effects from a low CNT concentration (5 µg/mL) could be recognized as a typical pharmacodynamic criterion of NOAEL for CNTs, similar to the sub-clinical effects of traditional lipophilic agents with mitochondrial mechanisms reported in the literature. As mentioned in the introduction of this work, CNTs can modulate mitochondrial function at a critical concentration of 10 µg/mL [25].

In this sense, it should be pointed out that the biocompatibility/toxicity relationship of the tested CNT family may be important in predicting the no-observed-adverse-effect level. This is not only limited to considerations of dosimetry in terms of concentration, but also applies to the influence of the physicochemical nanodescriptors of the tested CNTs (Raman nanodescriptors). Thus, the prediction of uncertainty factors or encrypted information in the CNT structure, such as new Raman spectra nanodescriptors, could be used to model the dosimetric criteria anticipated to be without an increased risk for CNT adverse effects [33,34].

### 3.2. Discussion of Computational Results

Discussion of the PTML computational study. LM produced poor results (mean *R*^2^_ts_ = 0.356; mean RMSE_ts_ = 0.0954), showing that the relationship between the input moving averages (MAs) of the CNT Raman SG spectral moment and the mitochondrial oxygen flow is not a linear one, or that these features did not include enough information to model this relationship. Starting with the non-linear methods, such as NN and RF, the performance of the regression model improved. Thus, NN provided a mean *R*^2^_ts_ of 0.672 and a mean RMSE_ts_ of 0.0681. The best NN model (split 3) was a single-hidden layer with 10 neurons, a structure of 8-10-1 (eight inputs, 10 neurons in one hidden layer, one output), a weight decay of 0.001, and an *R*^2^_ts_ of 0.739 and an RMSE_ts_ of 0.0613 [31].

**Figure 4:**
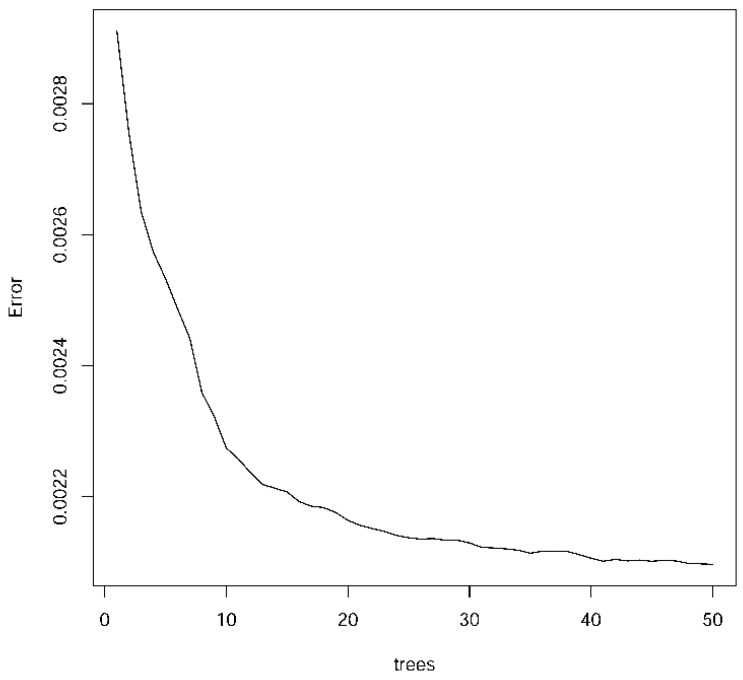
RF error with the number of trees for regression models.

**Figure 5:**
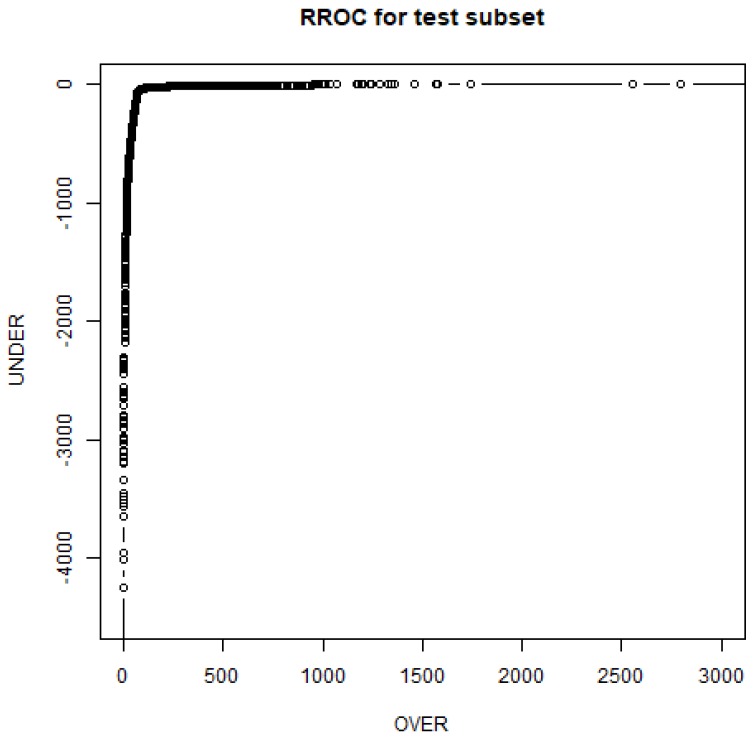
Regression receiver operator characteristic (RROC) curves for RF best model (test subset).

The RF predictor significantly improved the regression performance for mitochondrial oxygen flow: a mean *R*^2^_ts_ of 0.856 and a mean RMSE_ts_ of 0.0452 were obtained. Compared with the NN statistics, *R*^2^_ts_ increased by 0.184 and RMSE_ts_ decreased by 36.6%. The best RF model (split 2) has 50 trees, an *R*^2^_ts_ of 0.863 and an RMSE_ts_ of 0.0461. The variation in the RF error with the number of trees is shown in Figure 4. The model could be downloaded from the free online repository [31] and it could be used for future predictions or may be included in future R applications. The use of all initial 34 features had no important improvements in the regression performance with mean values for LM, NN and RF of 0.356, 0.601 and 0.857 (see all details online [31]). This means that the other features obtained no useful information to improve the current model. In order to check the model quality, the regression receiver operator characteristic (RROC) curve [35] for the test subset is shown in Figure 5. The RROC demonstrates the performance of the RF model. 

## 4. Materials and Methods 

### 4.1. General Workflow

The aims of this paper are:

(a) The measurement of the CNT effect on the mitochondrial oxygen mass flux with polarography;
(b) The definition and calculation of the matrix trace invariants (*Tr_k_*) of SG transforms of Raman spectra for a series of CNTs for the first time;
(c) The use of the *Tr_k_* values as input to seek new PTML models able to predict CNTs’ effect on mitochondrial oxygen mass flux.

**Figure 6:**
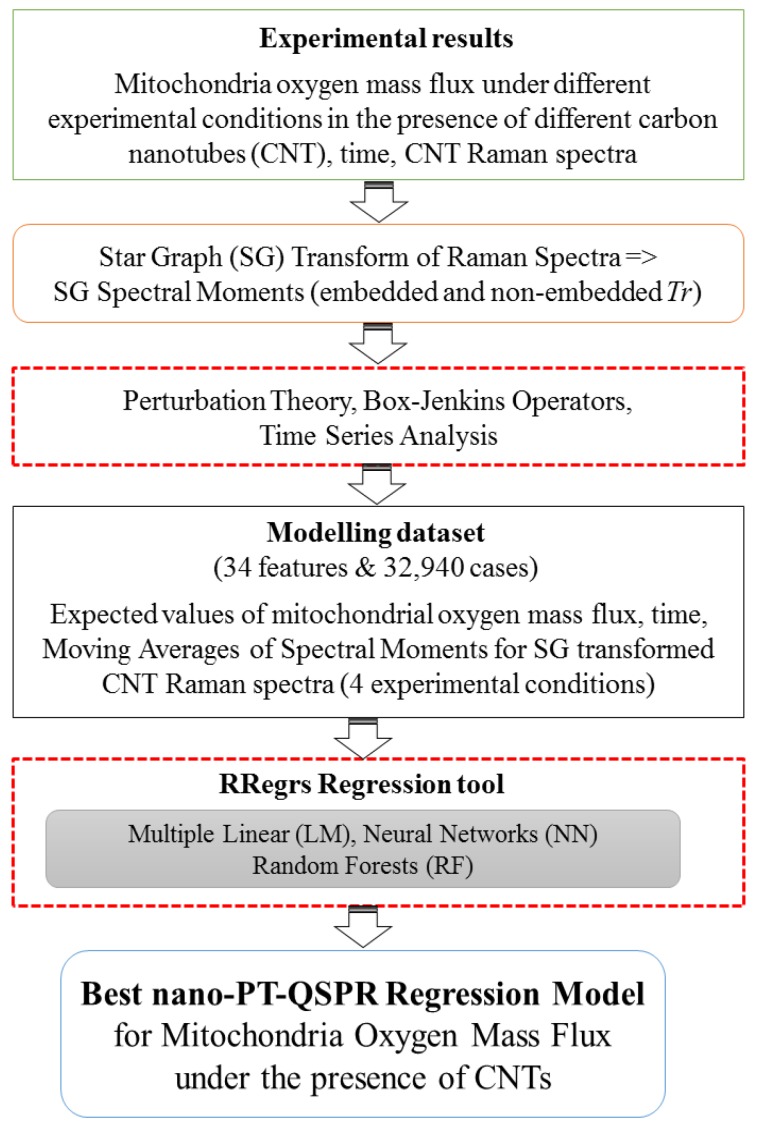
General workflow for the machine learning analysis.

Thus, the following steps were taken (see Figure 6):

(1) Experimental measurements of the mitochondria oxygen mass flux in the presence of different CNT types;
(2) Transformation of CNT’s Raman spectra into SG spectral moments;
(3) Calculation of the expected values of the mitochondria oxygen mass flux and the moving averages of the SG spectral moments under different experimental conditions;
(4) Search for the best regression PTML models using the RRegrs package in R (https://github.com/enanomapper/RRegrs/).

### 4.2. Experimental Methods

#### 4.2.1. General Procedures

In the Supplementary Information (SI) file, a detailed description of the following sections is included: sample preparation, reagents and solutions, Raman spectra recording, animal welfare, and isolation of rat liver mitochondria (RLM). The RLM were isolated by standard differential centrifugation according to the experimental procedures established in the literature [36].

#### 4.2.2. Monitoring Mitochondrial Oxygen Mass

The HRR method was used, along with Oroboros Instruments, DatLab Version 4.2.1.50 (Oxygraph-2k). This methodology included the use of a 2 mL glass chamber equipped with a magnetic stirrer. Mitochondrial O_2_ mass flux (pmol/seg) in the absence and presence of different carbon nanotubes (CNT-1 to CNT-9) was monitored and measured as the negative time derivative of an oxygen concentration (nmol/mL). The mitochondrial oxygen mass flux values were corrected for the small amount of back diffusion of oxygen from materials within the chamber, any leak of oxygen from outside the vessel, and oxygen consumed by the polarographic electrode [26,37,38].

With this in mind, the RLM isolated (1 mg protein/mL) were energized with 5 mM potassium succinate (plus 2.5 μM rotenone) in a standard incubation medium, consisting of 125 mM sucrose, 65 mMKCl, 2 mM inorganic phosphate (K_2_HPO_4_) and 10 mM potassium hydroxide-2-[4-(2-hydroxyethyl)piperazin-1-yl]ethanesulfonic acid (HEPES-KOH) pH 7.4 at 20 °C in a standard respiration medium. The experimental approach was calibrated using the oxygen content of an air saturated medium [38]. All the aforementioned steps were performed by the pre-incubation of isolated rat liver mitochondria with 5 µg/mL for all the CNT-treated groups. This level of concentration was considered based on the NOAEL criteria mentioned in the introduction of this work. For this instance, 5 µg/mL of the CNT concentration is the corrected half value of the CNT concentration (10 µg/mL) used by Ma et al., who promoted an incipient colloid–osmotic swelling or low induction of mitochondrial permeability transition pores, noticeably detected by Raman spectroscopy [15,16]. The total number of the collected data points was 32,940. The data obtained from the mitochondrial oxygen mass study in the presence of CNTs and the CNT spectra were used to search for a theoretical model which predicts mitochondrial oxygen mass in the presence of new CNTs.

### 4.3. Computational Methods

#### 4.3.1. Trace Invariants of Raman Spectra

One of the objectives of this work is to develop a mathematical model able to predict the biological effect of CNTs using as an input the information extracted from Raman spectra. Thus, a new type of parameters is proposed, calculated by the application of a Star Graph (SG) transform to the Raman spectra. The SRN transform method, which has been recently introduced and published by our group [17], uses graphs and network theory tools, and is different from a classic Fourier transformation. The transform technique used herein converts the Raman spectra values into sequences of characters and creates the corresponding SG of this signal. The SG of any sequence/signal may be constructed using the S2SNet tool [39]. To construct the SG transform of a Raman spectrum, the latter should be split into intervals of 100 units, from 0 to 1800. As a result, the maximum number of SG branches is 18 and corresponds to characters from “a” to “r”. Subsequently, the adjacency matrix **A** should be constructed for this sequence of characters (spectral sequence). The matrix **A** was enriched by adding the information about the recurrence to the same type of term in the spectral sequence. After this step, the matrix **A_e_** was obtained, with recurrence information embedded (**e**). As a result, different invariants from the matrices **A** and **A_e_** can be calculated. In this work, the matrix trace values (*Tr_k_* and *Tre_k_*; *k* = 0–5) were calculated, also known as the spectral moments [39] of the matrices **A** and **A_e_** obtained after the SG transformation of the Raman spectra. Our hypothesis is that these spectral moments encode useful structural information that can be responsible for biological activity and further predictive studies (see Figure 1).

#### 4.3.2. PTML Model

The current section describes the application of the algorithm of PTML heuristic models [30] in order to study the effect of different CNTs on the mitochondrial oxygen mass flux under different experimental conditions. The general equation of a linear PTML heuristic model could be described by Equation (1).

(1) $$J_{m}{\left(O_{2}\right)}_{pred}=a_{0}+a_{1}\cdot J_{m}{\left(O_{2}\right)}_{expected}+a_{2}\cdot f\left(t\right)+\overset{5}{\underset{k=0}{\sum }}b_{k}\cdot \Delta Tr_{k}\left(c_{j}\right)+\overset{5}{\underset{k=0}{\sum }}c_{k}\cdot \Delta Tre_{k}\left(c_{j}\right)$$

where *J_m_(O_2_)_pred_* is the predicted mitochondrial oxygen mass flux. The term *J_m_(O_2_)_expected_* = <*J_m_(O_2_)*> is the average value of *J_m_(O_2_)* for different subsets of experimental conditions (expected value of *J_m_(O_2_)*). The other input values *ΔTr_k_* and *ΔTre_k_* are the moving average (MA) operators. The MA operators *ΔTr_k_(c_j_)* = *Tr_k_* − <*Tr_k_(c_j_)*> and *ΔTre_k_(c_j_)* = *Tre_k_* − <*Tre_k_(c_j_)*> quantify the deviation of *Tr_k_* or *Tre_k_* of the Raman spectra for a specific CNT from the expected average values <*Tr_k_(c_j_)*> or <*Tre_k_(c_j_)*> measured for all Raman spectra, recorded for all CNTs with the specific experimental condition (*c_j_*). The symbols *Tr_k_* refer to traces calculated from linear graphs of the Raman spectra and the symbol *Tre_k_* for graphs with recurrence information embedded. The coefficients *a_k_*, *b_k_*, and *c_k_* are the linear coefficients of the equation.

#### 4.3.3. Model Dataset

The experimental data for the mitochondrial oxygen mass flux (*J_m_*) in the presence of CNTs are available as a FigShare repository [31]: 32,940 cases of *J_m_(O_2_)*, CNT1-9, CNTtype, Replicate (rep), Function_type (chemical modification of CNT), Solvent and time (*t*). *J_m_*(O_2_) was measured under four types of experimental conditions (c) such as Replicate (rep), CNTtype, Functiontype (chemical modification of CNT), and Solvent. Replicate (rep) has two values: 0 or 1, for non-replicated and replicated experiment. CNTtypes are multi-walled carbon nanotubes (MWCNT), mixed single walled/double-walled carbon nanotubes (SW+DWCNT), and single-walled carbon nanotube (SWCNT). CNT type is “0” when the assay is a control assay with a blank solution with a CNT concentration equal to 0. The values of the solvent condition are H_2_O and dimethyl sulfoxide (DMSO). The CNT Function types could have three values: 0 (none), COOH and OH. The average of the SG spectral moments under the experimental conditions are presented on the FigShare platform [31]. The final dataset, used to find the best prediction model is made up of 32,940 cases and 34 input features.

#### 4.3.4. PTML Regression Predictors

The raw dataset was normalized and the training and test sub-sets were obtained using 10 splits: 75% training sets (train) and 25% test sets (test) using an *R* script available online [40]. The regression PTML models were searched with RRegrs, an *R* integrated framework that provides ten linear and non-linear regression models [41,42]. The selection of the models used the criteria of the *R*_ts_ values (regression coefficient for test subset) and the RMSE_ts_ (root-mean-square error) corresponding values.

Three types of regression methods of RRegrs were used: multiple linear regression (LM), neural networks regression (NN) [43], and random forest (RF) [44]. Thus, the correlated features were removed using the parameters of RRegrs. For NN and RF, a study of the method parameters was performed. A modified version of RRegrs (batchRRegrs: https://github.com/cafernandezlo/batchRRegrs) was used on the BioCAI HPC platform from the University of A Coruna (A Coruña, Spain). The batchRRegrs default values of parameters were generally employed (https://github.com/cafernandezlo/batchRRegrs/blob/master/batchRRegrs/batchRRegrs.R). The default optimizations used the *tuneGrid* parameter of the caret training method:

- *NNreg* function used a grid for 200, 300 and 400 neurons in the hidden layer (*.size*) and a decay of 0, 0.01, 0.2, 0.1 (*.decay*) (*method* = ‘nnet’);
- *RFreg* function used 1500 trees (*ntree* = 1500 for *method* = ‘rf’).

An additional number of neurons were tested for NN (1, 5, 10 and 15) and different decay values (0.001 and 0.005). Moreover, an additional number of trees in RF were tested: 5, 10, 20, 30, 40, 50, 100, and 500. The best RF model should use the lowest number of trees and best statistics. The results presented for NN and RF models the parameters for the best models.

The criteria to find the best model apply the RRegrs methodology: maximum *R*^2^_ts_ and minimum RMSE_ts_. The plots were obtained with custom *R* scripts. The best regression model which predicts mitochondria oxygen mass flux in the presence of CNTs is available online [31] in order to be used for future predictions. The regression receiver operator characteristic (RROC) curve was constructed using the algorithm from reference [35]. The RROC presented the over-estimation (OVER) against the under-estimation (UNDER). Thus, the curve was drawn by adjusting a shift (a constant that was added or subtracted) for the predictions. This shift is similar to the threshold in the case of classifications.

## 5. Conclusions

The current study presented a mixture of experimental and predictive methodologies to study the effect of different CNTs on the mitochondrial oxygen mass flux. The experimental results showing non-significant respiratory effects from low CNT concentrations (5 µg/mL) could be recognized as a typical pharmacodynamics criterion of NOAEL for CNTs. In this context, the information encrypted in the Raman spectra of CNT structures can be used as novel nanodescriptors to model the complexity of the dosimetric criteria, as no adverse mitochondrial respiratory effect level (or normal O_2_ flux) was found. The *R* object model and an *R* script are available online at https://dx.doi.org/10.6084/m9.figshare.3545561.

These results show that the SG transform of CNT Raman spectra contains important information, as new CNT nanodescriptors can be combined to provide a prediction model under the experimental conditions over time for the mitochondria oxygen mass flux under the presence of specific CNTs. These in silico results indicate that this methodology can be employed for massive, virtual-based, raw data for Raman spectroscopy in order to make regulatory decisions in the biomaterial sciences.

## Figures and Tables

**Table 1:** Predictive model based on Machine Learning and Perturbation Theory (PTML) statistics for the evaluation of mitochondrial oxygen flow modifications due to CNTs (10 random splits for each method).

Regression Method	Statistics	Training		Test	
		*R*^2^_tr_	RMSE_tr_	*R*^2^_ts_	RMSE_ts_
Linear Multi-regression (LM)	Mean	0.358	0.0959	0.356	0.0954
	Min	0.349	0.0954	0.340	0.0932
	Max	0.363	0.0966	0.384	0.0969
Neural Network (NN)	Mean	0.645	0.0709	0.672	0.0681
	Min	0.626	0.0697	0.620	0.0613
	Max	0.659	0.0727	0.739	0.0738
Random Forest (RF)	Mean	0.855	0.0455	0.856	0.0452
	Min	0.851	0.0451	0.853	0.0431
	Max	0.858	0.0462	0.863	0.0461

## Supplementary Materials

The following are available online at http://www.mdpi.com/2079-4991/7/11/386/s1, Table S1. Properties of CNT families, Figure S1. Raman spectra for carbon nanotubes used in the present study, Figure S2. Control profile of mitochondrial oxygen mass flux of isolated rat liver mitochondria (Y2: Red curve).
