# Supplementary material for: Carbon Nanotubes’ Effect on Mitochondrial Oxygen Flux Dynamics: Polarography Experimental Study and Machine Learning Models using Star Graph Trace Invariants of Raman Spectra
Source: Nanomaterials (Basel). 2017 Nov 11;7(11):386. doi: 10.3390/nano7110386 (PMC5707603; doi:10.3390/nano7110386)
Supplement: Supplementary file 1 [file nanomaterials-07-00386-s001.pdf]

Supporting Information

Carbon Nanotubes Effect on Mitochondrial Oxygen Flux Dynamics: Polarography Experimental Study and Machine Learning Models using Star Graph Trace Invariants of Raman Spectra

Michael González-Durruthy [1], Jose M. Monserrat [1,*], Bakhtiyor Rasulev [2], Gerardo M. Casañola-Martín [3], José María Barreiro Sorrivas [4], Sergio Paraíso-Medina [5], Víctor Maojo [5], Humberto González-Díaz [6,7], Alejandro Pazos [8,9] and Cristian R. Munteanu [8,9,*]

1 Institute of Biological Science (ICB), Federal University of Rio Grande, Rio Grande, RS 96270-900, Brazil; gonzalezdurruthy.furg@gmail.com
2 Department of Coatings and Polymeric Materials, North Dakota State University (NDSU), Fargo, ND, USA; bakhtiyor.rasulev@ndsu.edu
3 Department of Systems and Computer Engineering, Carleton University, Ottawa, ON K1S 5B6, Canada; gmaikelc@gmail.com
4 Computer Science School (ETSIINF), Polytechnic University of Madrid (UPM), Calle de losCiruelos, Boadilla del Monte, 28660 Madrid, Spain; jmbarreiro@fi.upm.es
5 Biomedical Informatics Group, Artificial Intelligence Department, Polytechnic University of Madrid, Calle de los Ciruelos, Boadilla del Monte, 28660 Madrid, Spain; sparaiso@infomed.dia.fi.upm.es (S.P.-M.), vmaojo@fi.upm.es (V.M.)
6 Department of Organic Chemistry II, University of the Basque Country UPV/EHU, 48940 Leioa, Biscay, Spain; gonzalezdiazh@yahoo.es
7 IKERBASQUE, Basque Foundation for Science, 48011 Bilbao, Biscay, Spain
8 INIBIC Institute of Biomedical Research, CHUAC, UDC, 15006 Coruña, Spain; apazos@udc.es
9 RNASA-IMEDIR, Computer Sciences Faculty, University of Coruña, 15071 Coruña, Spain
* Correspondence: josemmonserrat@gmail.com (J.M.M.); c.munteanu@udc.es (C.R.M.); Tel.: +34-981-167-000 (ext. 1302) (C.R.M.)

Sample preparation

Reagents and solutions

Sucrose, ethylene glycol-bis (β-aminoethyl)-*N,N,N′,N′*-tetraacetic acid (EGTA), KCL, potassium succinate (plus 2 μM rotenone), K_2HPO_4, piperazine-*N′*-2-ethanesulfonic acid (Hepes-KOH). All the other reagents were commercial products of the highest purity grade available. For the mitochondrial oxygen mass flux test, the pristine and functionalized carbon nanotubes (MWCNT, [SWCNT+DWCNT]–OH, MWCNT–OH, MWCNT–COOH, SWCNT–COOH) were dissolved in dimethyl sulfoxide (DMSO) and Milli-Q water in individual stock suspensions was prepared at 1 mg/ml. The CNT families (see Table SM01) were provided by Cheaptubes Company (http://cheaptubes.com/shortohcnts.htm).

Table S1. Properties of CNT families.

CNT-properties			W_i (%)		D_i(nm)		L_i	P_i	C_i
n	type	function	min	max	min	max	(μm)	(%)	S cm^{-1}
1	MWCNT	-	-	-	8	8	0.5-2	>95	<1.5
2	Mixed-SW/DWCNT	OH	0	3.96	1	4	0.5-2	>95	<1.5

3	MWCNT	OH	0	3.86	1	8	0.5-2	>95	<1.5
4	MWCNT	OH	3	4	10	20	0.5-2	>95	<1.5
5	MWCNT	OH	1	1.06	30	50	0.5-2	>95	<1.5
6	MWCNT	COOH	0	0.73	30	50	0.5-2	>95	<1.5
7	MWCNT	COOH	3	4	10	20	0.5-2	>95	<1.5
8	SWCNT	COOH	0	2.73	1	4	0.5-2	>95	<1.5
9	MWCNT	COOH	0	3.86	1	8	0.5-2	>95	<1.5

MWCNT = Multiple-Walled, SWCNT = Single-Walled, SW/DWCNT = DWCNT + SWCNT mixture, W_i(%) = Functional groups(OH, COOH)/carbon atoms ratio (%); the properties of the ith Carbon Nanotube (CNT) are D_i = CNT outer diameter, L_i = CNT Length, P_i = Purity, C_i = Electric conductivity.

Raman Spectra

Individual carbon nanotubes Raman spectra were obtained by using a Renishaw Micro-Raman Spectroscopy System with a laser excitation wavelength of 514 nm (2.33 eV, see Fig. SM01). All reactions were quenched to room temperature before Raman spectra were obtained to identify the corresponding peaks at 1,580 cm^{-1} (G band of graphite) and the second peak at 1,350 cm^{-1} (D band of defects), according to the presence of the CNT defects generated by covalent functionalization (CNT oxidation) in the graphite structure (oxidized CNT with OH and COOH functional groups).

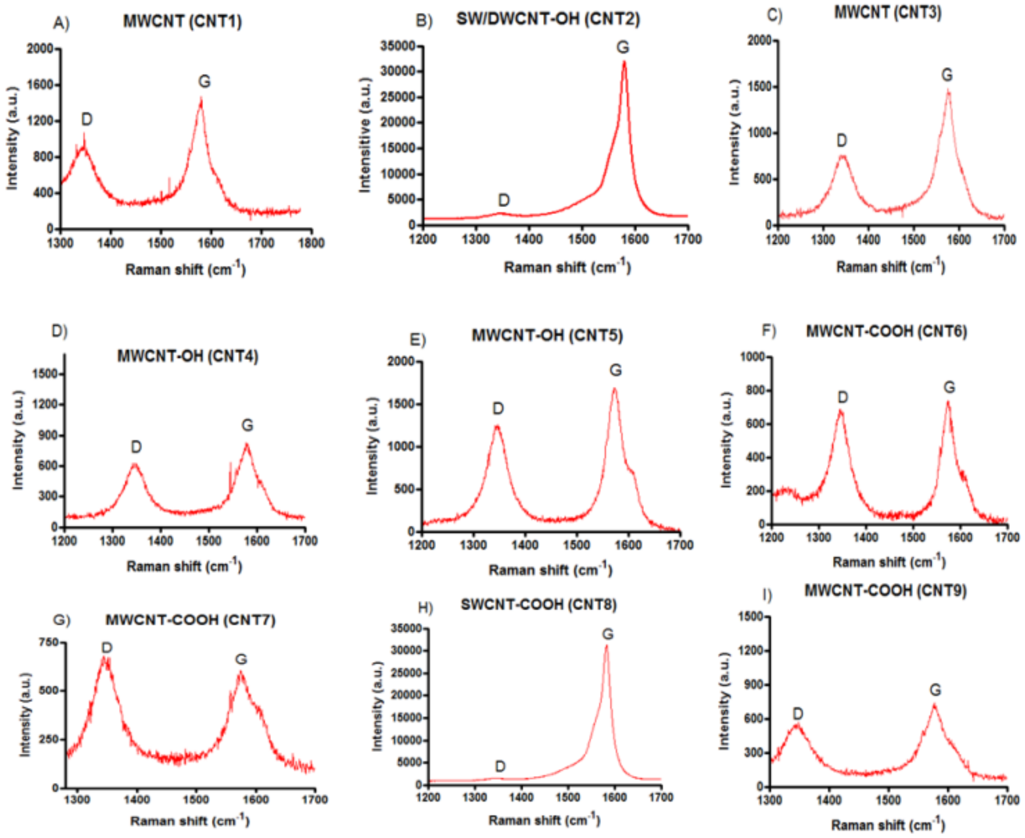

Figure S1. Raman spectra for carbon nanotubes used in the present study.

Animal Welfare

Male *Wistar* rats (4-month old; approx. 150 g) received food and water *ad libitum*. They were kept in plastic cages with wire tops in a light-controlled room (12:12 h light–dark cycle) at 22±3 °C before starting the biochemical assays according to the Directive 2010/63/EU of the European

Parliament and of the Council on the protection of animals used for scientific purposes, and following the Alternative Toxicology based on the "3R principle" (reduction, refinement and replacement by in vitro testing); these procedures were also approved by the Institutional Animal Care and Use Committee of the School of Pharmaceutical Sciences of Ribeirão Preto (CEUA-FCFRP) (license and registration number: 01.0263.2014).

Isolation of rat liver mitochondria (RLM)

Rat Liver Mitochondria (RLM) were isolated by standard differential centrifugation according to the experimental procedures established in the literature [30]. For the standard incubation procedure, the isolated mitochondria were energized with 5 mM potassium succinate (plus 2.5 μM rotenone) in a standard incubation medium consisting of 125 mM sucrose, 65 mM KCl, 2 mM inorganic phosphate (K_2HPO_4) and 10 mM HEPES-KOH pH 7.4 at 30 °C [30].

Control profile of mitochondrial oxygen mass flux of isolated RLM

In addition, we included Figure SM02 that depicts a control profile of mitochondrial oxygen mass flux of isolated rat liver mitochondria (Y2: Red curve) showing the inhibition of ATP synthesis or V3 state (ADP-dependent respiration) promoted by pre-incubation with CATR (ANT-1 classical inhibitor) represented as an absence of transient increments of O2 flux after the addition of small pulses of ADP. The CATR, ADP pulses and the uncoupling agent (FCCCP) were added where indicated by the arrow.

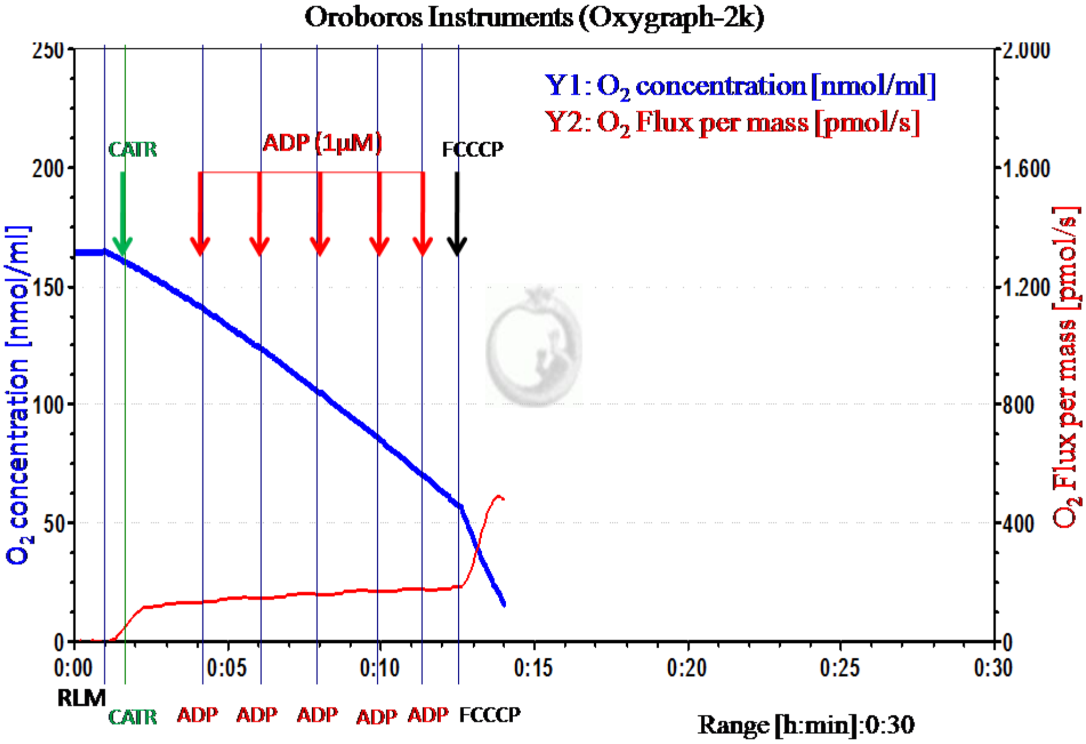

Figure S2. Control profile of mitochondrial oxygen mass flux of isolated rat liver mitochondria (Y2: Red curve).
